# A temperate super-Jupiter imaged with JWST in the mid-infrared

**DOI:** 10.1038/s41586-024-07837-8

**Published:** 2024-07-24

**Authors:** E. C. Matthews, A. L. Carter, P. Pathak, C. V. Morley, M. W. Phillips, S. Krishanth P. M., F. Feng, M. J. Bonse, L. A. Boogaard, J. A. Burt, I. J. M. Crossfield, E. S. Douglas, Th. Henning, J. Hom, C.-L. Ko, M. Kasper, A.-M. Lagrange, D. Petit dit de la Roche, F. Philipot

**Affiliations:** 1 Max Planck Institute for Astronomy, Heidelberg, Germany; 2 Space Telescope Science Institute, Baltimore, MD USA; 3 Department of SPASE, Indian Institute of Technology Kanpur, Kanpur, India; 4 Department of Astronomy, University of Texas at Austin, Austin, TX USA; 5 Institute for Astronomy, University of Edinburgh, Royal Observatory, Edinburgh, UK; 6 Steward Observatory and Department of Astronomy, Tucson, AZ USA; 7 Shanghai Jiao Tong University, Shanghai, People’s Republic of China; 8 ETH Zurich, Institute for Particle Physics & Astrophysics, Zurich, Switzerland; 9 Max Planck Institute for Intelligent Systems, Tübingen, Germany; 10 Jet Propulsion Laboratory, California Institute of Technology, Pasadena, CA USA; 11 Department of Physics and Astronomy, University of Kansas, Lawrence, KS USA; 12 European Southern Observatory, Munich, Germany; 13 LESIA, Observatoire de Paris, Université PSL, CNRS, Meudon, France; 14 Department of Astronomy, University of Geneva, Versoix, Switzerland

**Keywords:** Exoplanets, Atmospheric chemistry

## Abstract

Of the approximately 25 directly imaged planets to date, all are younger than 500 Myr, and all but six are younger than 100 Myr (ref. ^1^). Eps Ind A (HD209100, HIP108870) is a K5V star of roughly solar age (recently derived as 3.7–5.7 Gyr (ref. ^2^) and ${3.5}_{-1.3}^{+0.8}$ Gyr (ref. ^3^)). A long-term radial-velocity trend^4,5^ and an astrometric acceleration^6,7^ led to claims of a giant planet^2,8,9^ orbiting the nearby star (3.6384 ± 0.0013 pc; ref. ^10^). Here we report JWST coronagraphic images which reveal a giant exoplanet that is consistent with these radial and astrometric measurements but inconsistent with the previously claimed planet properties. The new planet has a temperature of approximately 275 K and is remarkably bright at 10.65 and 15.50 µm. Non-detections between 3.5 and 5.0 µm indicate an unknown opacity source in the atmosphere, possibly suggesting a high-metallicity, high carbon-to-oxygen ratio planet. The best-fitting temperature of the planet is consistent with theoretical thermal evolution models, which were previously untested at this temperature range. The data indicate that this is probably the only giant planet in the system, and therefore we refer to it as b, despite it having significantly different orbital properties than the previously claimed planet b.

## Abstract (web summary)

We report on JWST images of a temperate super-Jupiter in the system Epsilon Ind A—a K5V star at a distance of just 3.6 pc—in the mid-infrared.

## Main

**Fig. 1:**
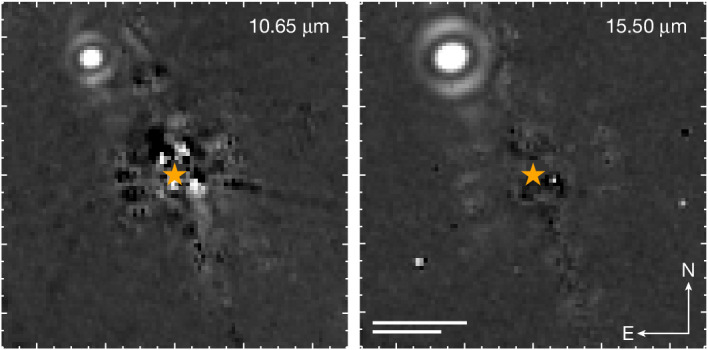
Point source detected in JWST/MIRI coronagraphic images of Eps Ind A. The target was observed at 10.65 and 15.50 µm. Starlight was removed with RDI. Only the central portion of the field of view is shown. The stellar position is marked with an orange star. A bright point source was detected in the upper left corner of these images at a projected separation 4.11″ (15.0 au at the distance of Eps Ind A). Top scale bar, 2.75″ = 10.0 au; bottom scale bar, 2″ = 7.28 au.

We observed Eps Ind A with the mid-infrared instrument (MIRI)^11^ coronagraph onboard the James Webb Space Telescope (JWST) on 3 July 2023 using two narrowband filters (10.65 and 15.50 µm). Figure 1 shows a bright point source detected in the north-east quadrant at a separation of 4.11″. This is the opposite quadrant than expected based on previous orbital solutions^2,8,9^. The source is unresolved, has apparent magnitudes 13.16 and 11.20 mag at 10.65 and 15.50 µm, and is consistent with a cold, Jupiter-sized object at the host star distance. We ruled out chance-aligned background objects and recovered the companion at a low signal-to-noise ratio in archival observations, confirming that it is physically associated with the host star. The source has significantly different orbital properties than the literature solutions for Eps Ind Ab, but we conclude that it is probably the only massive planet in the system and is responsible for the previously observed radial velocity and astrometric acceleration of the host star.

Owing to the unexpected location of the source, we first assessed whether it could be a chance-aligned background object. This is statistically unfavoured. Source-counting studies with the MIRI broadband filters^12^ indicate an expected background density of approximately 45 sources per square degree at least as bright as the point source, corresponding to just a 0.027% likelihood of finding such a source within 5″ of the target. Although occurrence rates for giant, long-period planets are low^13,14^, the probability of a chance-aligned background object is much lower. A background contaminant was further constrained based on archival data. The extremely high proper motion of Eps Ind A (4,708.15 ± 0.13 mas yr^−1^; ref. ^10^) means that the background location is unobstructed by the star in sufficiently old data. We studied previous observations of Eps Ind A, and no stationary background contaminant consistent with the point source was detected. A particularly strong constraint comes from archival observations by the infrared array camera (IRAC) onboard Spitzer^15^, which rules out companions brighter than 16.0 mag at 8 µm. The point source is 13.16 mag at 10.65 µm. Further, the source is unresolved, and most galaxies of this brightness would be spatially resolved at MIRI wavelengths. It is unlikely that a stationary background object would have evaded detection in all archival observations while also reproducing the candidate planet properties. A Solar System source (for example, an asteroid) can be excluded as it should have moved between the F1065C and F1550C observations. A transient source (for example, a burst) cannot be excluded based on archival data alone but is strongly disfavoured statistically.

**Fig. 2:**
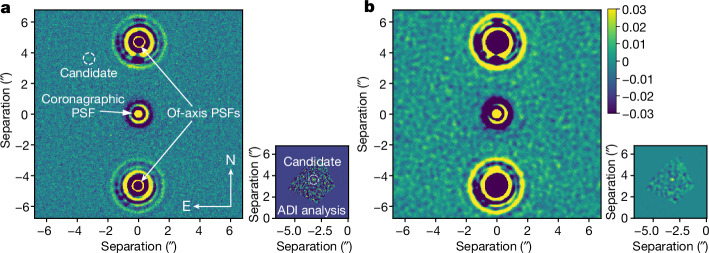
Point source detected at a consistent location in VISIR/NEAR images of Eps Ind A. **a**,**b**, Co-added images (main panels) and PCA results for a small patch around the expected target location (insets). Panel **b** shows the images in **a** after convolution with a top-hat function.

We reanalysed archival data collected with the VISIR/NEAR instrument^16,17^ at the Very Large Telescope (VLT). These coronagraphic observations of Eps Ind A were collected over three nights during September 2019 using a broadband filter spanning approximately 10.0–12.5 µm. At approximately 15 au, a planet would have an orbital period of at least several decades, corresponding to little orbital motion in the approximately 3.8 yr between the VISIR and JWST observations, whereas a background source would have shifted by 18'' over this time baseline. Figure 2 shows the VISIR/NEAR observations. A faint point source at a signal-to-noise ratio of approximately 3 was observed. It is consistent with the expected flux and location of the JWST point source. We concluded that this is an archival redetection of the same object, which confirms its common proper motion and conclusively demonstrates that it is a planet (hereafter Eps Ind Ab).

**Fig. 3:**
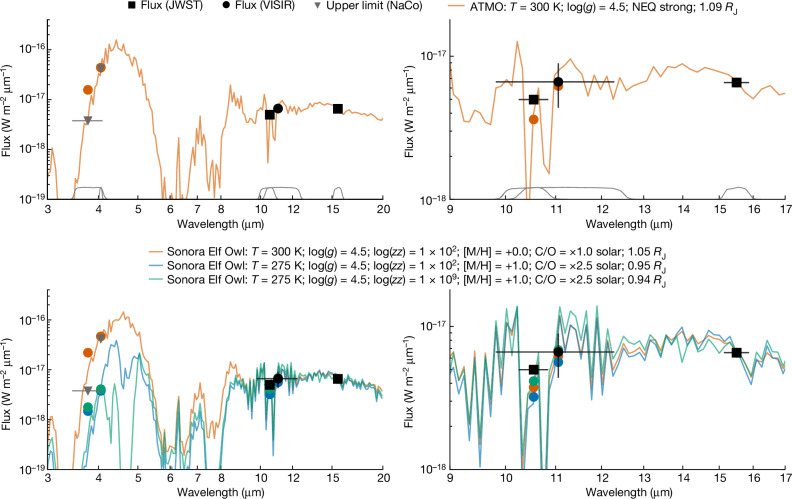
Eps Ind Ab is consistent with theoretical atmosphere models with suppressed 3.5–5.0 µm flux. Measured photometry (squares and circles with uncertainties of 1*σ*) and 5*σ* upper limits (triangles), compared to out-of-the-box model spectra^24,27^ (coloured lines; circles indicate the integrated flux for each observed filter). Normalized filter profiles are overlaid as grey lines. Planet radii are scaled to match the measured 15.50 µm photometry, and labels indicate the temperature *T*, surface gravity *g*, log eddy diffusion coefficient (log(*zz*)), log-metallicity ([M/H]), and carbon-to-oxygen ratio (C/O). The ATMO models include non-equilibrium (NEQ) chemistry. A high metallicity and carbon-to-oxygen ratio are required to suppress the 3.5–5.0 µm flux to below the observed upper limit.

The measured mid-infrared photometry (10.65 and 15.50 µm) of Eps Ind Ab is consistent with atmosphere models for an approximately 300 K planet, as demonstrated in Fig. 3. However, these models typically predict significant emission between 3.5 and 5.0 µm. Archival observations by the NaCo instrument at the Very Large Telescope (VLT)^17,18^ failed to detect Eps Ind Ab. Atmospheric models predict a ‘window’ for flux at these wavelengths that is between absorption features from molecular species including CH_4_ and H_2_O (ref. ^19^). This is observed for cold brown dwarfs^20,21^ and expected for cold, giant planets but is in tension with NaCo upper limits (highlighted in Fig. 3). The NaCo non-detection suggests that there is another opacity source in the 3.5–5.0 µm region, such as molecular absorption or significant cloud or haze high in the atmosphere. Although some model grids explore cloudy scenarios^22,23^, these models require patchy clouds and the 3.5–5.0 µm flux is dominated by the cloud-free regions of the atmosphere. This, in turn, leads to a pronounced emission peak, similar to that seen in the ATMO models (Fig. 3). Sonora Elf Owl models^24^ with temperature 275 K, high metallicity ([M/H] = 1.0), high carbon-to-oxygen ratio (×2.5 solar) and strong disequilibrium chemistry^25^ (high log(*K*_*zz*_), where *K*_*zz*_ is the eddy diffusion coefficient), are compatible with all in-hand observational constraints. This model includes significant CH_4_, CO_2_ and CO absorption, which suppresses the 3.5–5.0 µm flux, and a modest ammonia absorption feature that reproduces the 10.65 µm flux. A high metallicity is somewhat surprising for a super-Jupiter planet; the C/O ratio is consistent with predictions for a planet formed by core accretion beyond the CO_2_ ice line^26^. Further photometric and spectroscopic characterization of the planet is crucial to confirm these preliminary indications of the atmospheric C/O ratio and to confirm whether chemistry or haze particles are the true cause of the suppressed 3.5–5.0 µm flux and allow a comparison to formation models.

To constrain the dynamical mass of Eps Ind Ab, we fitted a single-planet orbit to all published radial-velocity data (493 points from four facilities over 29 yr) for the two imaging epochs and to the Hipparcos–Gaia astrometry. This gave a mass of ${6.31}_{-0.56}^{+0.60}$ Jupiter masses (*M*_J_) and a semimajor axis of ${28.4}_{-7.2}^{+10}\,{\rm{au}}$. The fitted orbit is eccentric ($e={0.40}_{-0.18}^{+0.15}$) and observed at apastron, and this is able to explain all in-hand data. Although previous studies claimed the presence of a 3.0 ± 0.1 *M*_J_ planet with semimajor axis ${8.8}_{-0.1}^{+0.2}\,{\rm{au}}$ (refs. ^2,8,9^), based on the radial-velocity data and astrometric information, the imaged planet imparts a significant acceleration on the host star, and so it is clear that there is no inner exoplanet with the previously published parameters. Although the data do not exclude the possibility that there is a second planet in the system, Eps Ind Ab is the dominant accelerator of the star. Future works should explore why the previous radial-velocity analyses led to underestimated mass and semimajor axis constraints for this system, which may be due to overfitting or if there are, in fact, several planets in the system.

**Fig. 4:**
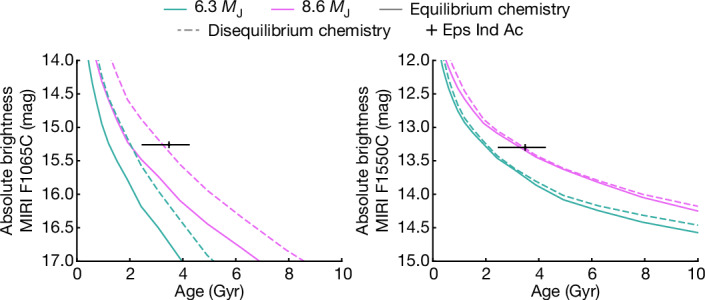
Comparison of Eps Ind Ab to evolutionary models. Cooling curves for a 6.3 *M*_J_ planet and an 8.6 *M*_J_ planet for models with equilibrium and with non-equilibrium chemistry^27^. The mid-infrared companion photometry is consistent with a more massive planet, although these models do not incorporate the high metallicity or carbon-to-oxygen ratio of the planet nor the correspondingly low 3.5–5.0 µm flux.

Evolutionary models predict a temperature of approximately 280 K and a luminosity log(*L/L*_⊙_) of approximately −7.2 for a 3.5 Gyr, 6.3 *M*_J_ planet^27^. Figure 4 shows evolutionary models^27^ for two planets: a 6.3 *M*_J_ object (matching the dynamical mass) and an 8.6 *M*_J_ planet (matching the observed photometry), considering both equilibrium and disequilibrium chemistry. Based only on the age and MIRI photometry for Eps Ind Ab, the planet is overluminous relative to the models. However, the extent of the flux suppression at 3.5–5.0 µm remains unclear, and this impacts the derived temperature of the planet. The best-fitting model in Fig. 3 incorporates this suppressed flux. It gives a temperature of 275 K, which is very close to the temperature predictions from evolutionary models. Further photometry between 3.5 and 5.0 µm is crucial for confirming the extent of flux suppression, better constraining the temperature of Eps Ind Ab and, thereby, allowing a more thorough comparison to evolutionary models.

Eps Ind Ab is the coldest exoplanet to have been directly imaged. It has a temperature close to that of the coldest field brown dwarf (WISE 0855)^21^. The system is also co-moving with a widely separated brown dwarf binary^28,29^, making it a particularly valuable laboratory for comparative studies of substellar objects with a shared age and formation location. The exoplanet detection highlights the power of using indirect evidence to target direct detection efforts. Even though the detected planet does not match the previously claimed properties for an exoplanet orbiting this star, the long-term radial-velocity information was crucial in motivating the value of imaging this target. The photometry of Eps Ind Ab suggests it has a high metallicity and carbon-to-oxygen ratio, and future works should aim to measure the planetary flux between 3.5 and 5.0 µm to determine the extent of flux suppression. Future works should also aim to expand the sample of cold exoplanets and determine whether other cold planets show the same low 3.5–5.0 µm flux, indicating high metallicity and high carbon-to-oxygen ratio. Two candidate companions to white dwarf stars^30^ and several upcoming JWST observations of nearby accelerating stars^31,32^ may mean that a small sample of cold, solar-age exoplanets can be assembled. The impact of atmospheric absorption is also critical when deriving mass detection limits, which are used to calculate occurrence rates, and when designing future observations of cold exoplanets. The bright flux and wide separation of Eps Ind Ab mean that the planet is ideally suited to spectroscopic characterization efforts, which may more accurately constrain its metallicity and carbon-to-oxygen ratio.

## Methods

### JWST observations and data reduction

We observed Eps Ind A with the JWST/MIRI coronagraphic imager^11,33,34^ on 3 July 2023 (JWST General Observer programme 2243; ref. ^35^) in a single sequence. Our observations consisted of science, reference and background images, as detailed in Extended Data Table 1. We collected images with two narrowband coronagraphic filters, F1065C and F1550C, which were integrated for 3,772 and 3,922 s, respectively. Each coronagraphic filter has a dedicated four-quadrant phase-mask coronagraph^36^ to suppress the stellar point spread function (PSF). Dedicated background observations of an empty patch of sky were made at the start of the sequence to allow subtraction of the ‘glow stick’ stray-light feature, first identified in MIRI commissioning^34^. For each filter and each of the science and reference targets, we collected background observations using the same integration as a single science/reference dither, and we repeated this process over two dither positions. We used the same empty field for all background observations.

**Extended Data Fig. 1:**
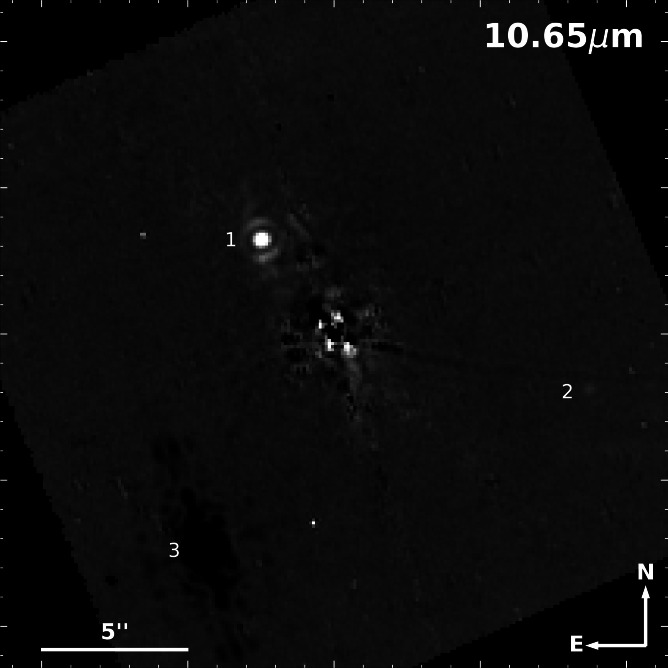
Full field-of-view JWST/MIRI coronagraphic images of Eps Ind A in the 10.65 µm filter. Two visual companions to Eps Ind A, and one visual companion to DI Tuc, are identified: (1) is Eps Ind Ab, (2) is the background star Gaia DR3 6412595290591430784 (also identified in the Spitzer 8 µm observations of the background field) and (3) is the background star Gaia DR3 6411654761473726464 in the field of the DI Tuc observations.

We collected PSF reference images of the star DI Tuc (HD211055) for reference differential imaging (RDI)^37^. We collected five reference images for each filter using the five-point small-grid dither technique^38,39^ to account for the imperfect pointing of the JWST (commissioning data suggest a pointing stability of approximately 5–10 mas; ref. ^40^). The integration times per dither position were 2,631 and 2,724 s for the F1065C and F1550C observations, respectively. These integrations were chosen to match the peak flux of the Eps Ind A observations at each dither position. In advance of the JWST observations, we also screened the reference star for faint companions to ensure it was suitable for differential imaging. We observed DI Tuc with the Spectro-Polarimetric High-Contrast Exoplanet Research instrument (SPHERE) at VLT on 13 April 2023 (programme 110.25BR, PI E.C.M.) and collected 140 images, each with detector integration time (DIT) = 0.837 s (total exposure time 117 s), in the broadband H filter (*λ* = 1.6255 µm and Δ*λ* = 0.291 µm). Images were dark- and flat-fielded and bad pixels corrected, and we then aligned all images based on the peak flux position and co-added across the stack of observations. No companions were detected anywhere in the SPHERE field of view (approximately 12″ × 12″). Two companions are in the Gaia catalogue and the JWST field of view but outside the SPHERE field of view (separations 6.5″ and 8.2″). These are sufficiently widely separated that they did not impact the PSF subtraction, though the brighter of these is clearly visible in the reduced data (Extended Data Fig. 1).

We used the spaceKLIP pipeline^41,42^ for the JWST data analysis. This pipeline provides various high-contrast imaging-specific functionalities for JWST data and, in particular, makes use of the jwst pipeline^43^ and the pyKLIP Python package^44^ for several key reduction steps. Our reduction follows the steps outlined in detail in ref. ^42^ and is briefly summarized here. We started from the stage 0 files (*uncal.fits) generated by the jwst pipeline. We performed ramp-fitting, calibrated the images from detector units to physical units, flat-fielded and subtracted the background images. We then subtracted the stellar PSF using RDI. We explored various parameters. The reductions shown in this work were made using principal component analysis (PCA) with a single subsection and a single annulus. Eight PCA modes were removed.

**Extended Data Fig. 2:**
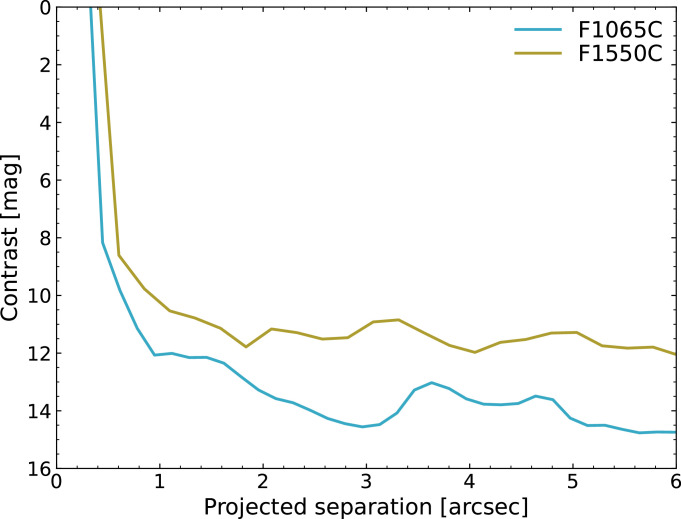
Contrast sensitivity of the MIRI observations. Curves are 5$\sigma$ sensitivities calculated with spaceKLIP for each coronagraphic filter.

This process provided excellent starlight suppression for the F1550C images. The contrast performance is provided in Extended Data Fig. 2. The stellar PSF is less well matched between the science and reference images for F1065C, probably due to the larger time baseline between these observations (the order of observations is F1065C reference, F1550C reference, F1550C science and F1065C science, as listed in Extended Data Table 1). This reduced the planet sensitivity at close separations, and some residual starlight is clearly visible in these images. The full field of view for the 10.65 µm images is shown in Extended Data Fig. 1, and the central portion of both images is included in Fig. 1. Three astrophysical point sources were detected at F1065C:

1. A bright source to the north-east of Eps Ind A was not previously known. This source was confirmed to be the exoplanet Eps Ind Ab in the current work.
2. A faint source to the west of Eps Ind A is a background star. This source corresponds to Gaia DR3 6412595290591430784 and was also detected in archival Spitzer/IRAC observations.
3. A negative source to the south-east of Eps Ind A is a widely separated companion to DI Tuc. It was subtracted in our RDI reduction. This source corresponds to Gaia DR3 6411654761473726464.

We determined the companion properties using the forward-model PSF fitting routine provided by pyKLIP and implemented in spaceKLIP. We generated a PSF template at the location of the companion using webbpsf_ext. We then fitted the location and intensity of this PSF to the data to measure the photometry and astrometry of the observations; these values are listed in Extended Data Table 2.

We also reduced the data using non-negative matrix factorization^45,46^ to verify our results. Non-negative matrix factorization involves decomposing an image matrix into two non-negative matrices, one has features and the other weights for reconstructing the original matrix. The features matrix extracted from the RDI PSF library was used to model the stellar PSF in the target image frames. PSF subtraction was then performed to image the exoplanet. We performed the non-negative matrix factorization reduction using all frames from the DI Tuc PSF library to construct components. For F1065C, this involved using 605 frames to construct 605 components. For F1550C, the number of frames and components was 65. We tried alternative approaches, including using the entire PSF library with fewer components and using a smaller PSF library with components selected using their closeness to the target frames (using the Euclidean norm), but using the entire library with the maximum number of components consistently produced the best images.

### Background constraints derived from archival data products

Our analysis of archival data provided tight constraints on a possible background contaminant. If the point source were an extragalactic contaminant, it would have no proper motion. It would be found 4.1″ to the north-east of the JWST Eps Ind A position, namely at right ascension 22 h 03 min 33.17 s and declination −56° 48′ 06.0″. For a background object, these absolute right ascension and declination coordinates would not change as Eps Ind A moves across the sky at a rate of 4.7″ yr^−1^ and would be well resolved from the star in sufficiently old archival data (allowing for a sufficient movement of the foreground star, relative to the background position). Even if a background contaminant were a distant object within the Milky Way, it would have a proper motion of at most a few milliarcseconds per year and at most a few tens of milliarcseconds over the approximately 2 decades spanned by the data products presented here. We searched the Gaia^10^ and 2MASS^47^ catalogues and reanalysed data from the IRAC and the multiband imaging photometer (MIPS) onboard Spitzer, but we did not identify any background sources consistent with the point source.

**Extended Data Fig. 3:**
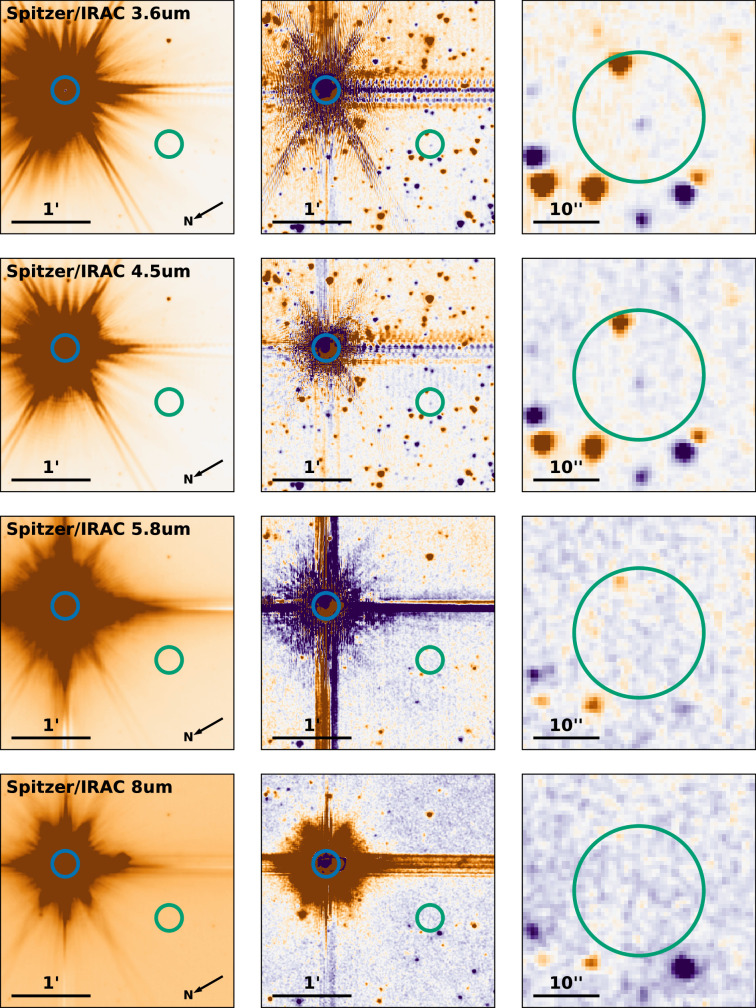
Spitzer IRAC images of Eps Ind A. The three columns show the science-ready Eps Ind A image from the Spitzer archive (left), an RDI processed image with Eps Eri subtracted from Eps Ind A (centre), and the RDI processed image zoomed at the JWST epoch position of Eps Ind A (right). For the RDI processed image, purple (negative) sources are in the field of the reference star, and orange (positive) sources are in the field of Eps Ind A. Blue and green circles have radii 10'', and are centred on the Spitzer and JWST epoch positions of Eps Ind A respectively. If Eps Ind Ab were a background object, it would be ~4'' from the JWST epoch position of Eps Ind A. This is well within the blue circle. One source is identified within this circle at 3.6 µm, 4.5 µm and marginally at 5.8 µm: this is a background star also identified in the JWST/MIRI images (see text for details).

Spitzer observations provide excellent background constraints and bracket the wavelengths of our JWST observations. Several Spitzer observations of Eps Ind A were carried out during 2004. Eps Ind A had moved approximately 90'' in the 19 yr between the Spitzer and JWST observations. Eps Ind A was observed with Spitzer/IRAC on 1 May 2004 (programme ID 90; PI M. Werner), with data collected in all four IRAC channels (nominally 3.6, 4.5, 5.8 and 8.0 µm). The star was used primarily as a PSF reference star for a programme studying the Eps Eri debris disk^15,48,49^. We reanalysed these data, starting from the science-ready mosaic files from the Spitzer Heritage Archive for each of the four wavelength channels. Even though the background position was well separated from the star at this epoch, there was still a significant signal in the PSF wings (Extended Data Fig. 3). We used the Eps Eri observations from the same programme as a PSF reference for RDI and for subtracting the stellar PSF wings, thereby allowing the best possible contrast at the background location. We used a grid-based approach to optimize the spatial (*x*, *y*) location and flux scaling of the Eps Eri images and, thereby, to provide the best match to the PSF wing structure of the Eps Ind images. We then subtracted this best-matching image from the Eps Ind data to produce the RDI-subtracted images. The science-ready images and RDI-subtracted images are shown in Extended Data Fig. 3. The position of Eps Ind A during the Spitzer and JWST epochs is highlighted. We calculated the flux limits based on the pixel variance in the RDI-subtracted image, with known sources masked, and validated our limits by performing aperture photometry on identified sources in the image.

**Extended Data Fig. 4:**
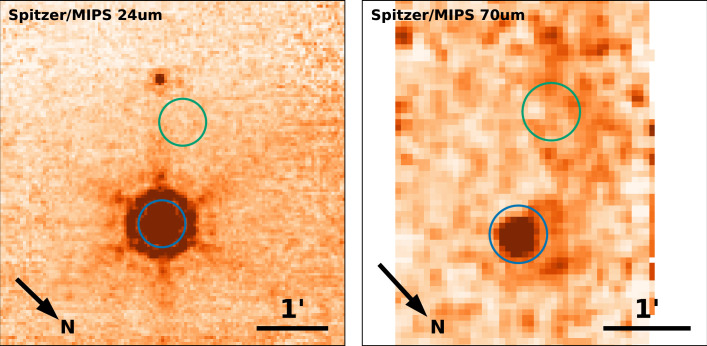
Spitzer MIPS 24µm and 70µm images of Eps Ind A. Blue and green circles have radii 20'', and are centred on the Spitzer and JWST epoch positions of Eps Ind A respectively; no sources are detected at the background position (the JWST epoch position of Eps Ind A). The 70 µm data cover only a narrow strip around the Eps Ind A location, and white regions at the edge of the box indicate regions outside the telescope field of view.

Eps Ind A was also observed with Spitzer/MIPS on 13 October 2004 (programme ID 41; PI G. Rieke)^50^. Data were collected in all three MIPS channels (nominally 24, 70 and 160 µm). We analysed the science-ready mosaic files from the Spitzer Heritage Archive. Eps Ind A is unsaturated in the MIPS images, and the JWST position is well resolved from the star (Extended Data Fig. 4) and within the field of view for the 24 and 70 µm channels. We calculated the flux upper limit based on the standard deviation of pixel values in a large box around the position of Eps Ind A, excluding pixels within 1 arcmin of the star. No sources were observed within 10″ of the JWST Eps Ind A position in the Spitzer IRAC 8.0 µm images or in any of the MIPS images. One source was identified approximately 9″ west of Eps Ind A in the 3.6, 4.5 and 5.8 µm images. This source was also identified in the JWST/MIRI images and is labelled 2 in Extended Data Fig. 1. The location and magnitude measurements correspond to that of the star Gaia DR3 6412595290591430784. This is a background object and is not associated with Eps Ind A.

**Extended Data Fig. 5:**
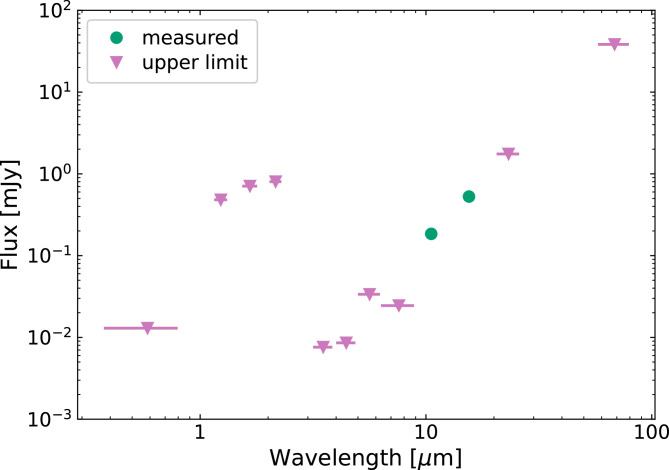
Spectrum (measured flux and upper limits) of the point source if it were a background object. These upper limits apply only if the point source were a stationary background at RA = 22:03:33.17 and Dec = −56:48:06.0. Constraints for the planetary case (which require high-contrast facilities to resolve a source that moves with same proper motion as the host star) are given in Fig. 3. Green circles indicate the measured JWST photometry, while pink triangles indicate upper limits from Gaia, 2MASS, Spitzer/IRAC and Spitzer/MIPS. Most stationary background contaminants would not reproduce the 10.65 µm and 15.50 µm flux while evading detection at other wavelengths.

A background spectrum for the point source is shown in Extended Data Fig. 5. This indicates the flux and upper limits that would apply to the object if it were a stationary background object or a background object that had moved by less than a few hundred milliarcseconds per year. That is, these upper limits do not apply to the planet, which has the same proper motion as Eps Ind A and was not present at this location in the background observations. The 8.0 µm images provide a particularly stringent constraint on the background scenario. We ruled out background objects to 16.07 mag at this wavelength, whereas the point source is 13.16 mag at 10.65 µm. A background object would have to be extremely red to be compatible with both measurements.

### Reanalysis of archival VISIR/NEAR data

We revisited archival VISIR/NEAR^51,52^ observations of Eps Ind A. We started from previously published reduced datacubes^16^, for which frames were calibrated and aligned. We removed low-quality frames, and then binned and spatially high-pass filtered the remaining frames. The NEAR sensitivity was optimized for the small separation (0.5″–3.0″) around the stars. Ground-based thermal infrared observations require fast chopping to quickly subtract the varying sky background and excess low-frequency detector noise. With NEAR, chopping was done using the deformable secondary mirror of the VLT, which provides a maximum chop throw of about 4.5″. The reduction with angular differential imaging (ADI) relies on subtracting images taken at a different field orientation, which leaves residuals of real sources in the azimuthal direction. As the separation of the imaged planet from the host star was close to the chop throw, these residuals masked the planet if ADI were used for data reduction. To avoid this effect, we simply co-added data without performing ADI, as the expected separation was well within the background-limited regime rather than the contrast-limited regime. We also performed a locally optimized combination of images (LOCI)^53^ PCA ADI analysis with three principal components in a small patch around the expected companion location. This helped to improve the planet signal and suppress the thermal background. In each case, we also convolved the final images with a top hat to enhance any true signals in the data. Both images are shown in Fig. 2. To confirm the source, we also looked at the companion position relative to the off-axis PSFs. We centred, aligned and derotated the frames relative to this position. We found that the planet signal was also present in the final averaged convolved frames relative to the off-axis PSFs, negating the possibility of the source being a speckle or a ghost from internal reflections. We assumed an error of half the full-width at half-maximum on the companion position, due to the high-spatial resolution and sampling of the NEAR. The planet was at a separation of 4.82 ± 0.16″ and at a position angle of 40–45° anticlockwise of north. To estimate the flux, we used planet injection and recovery tests. As the planet signal was limited by the thermal background, we put a conservative range of (4–8) × 10^−5^ on contrast and a range of 0.18–0.35 mJy on flux. These values are also included in Extended Data Table 2.

### Orbital constraints

Our goal in targeting Eps Ind A with JWST coronagraphic imaging was to detect a known, massive planet for which dynamical mass information was accessible. However, the imaged planet is inconsistent with the previously claimed planet in the system. The planet has a significantly larger mass and semimajor axis than the claimed Eps Ind Ab (refs. ^2,8,9^) and a strikingly different on-sky position than predicted. It is clear that the imaged planet imparts a significant, long-term, radial-velocity acceleration on the host star, although it was initially unclear whether this is the only planet in the system.

**Extended Data Fig. 6:**
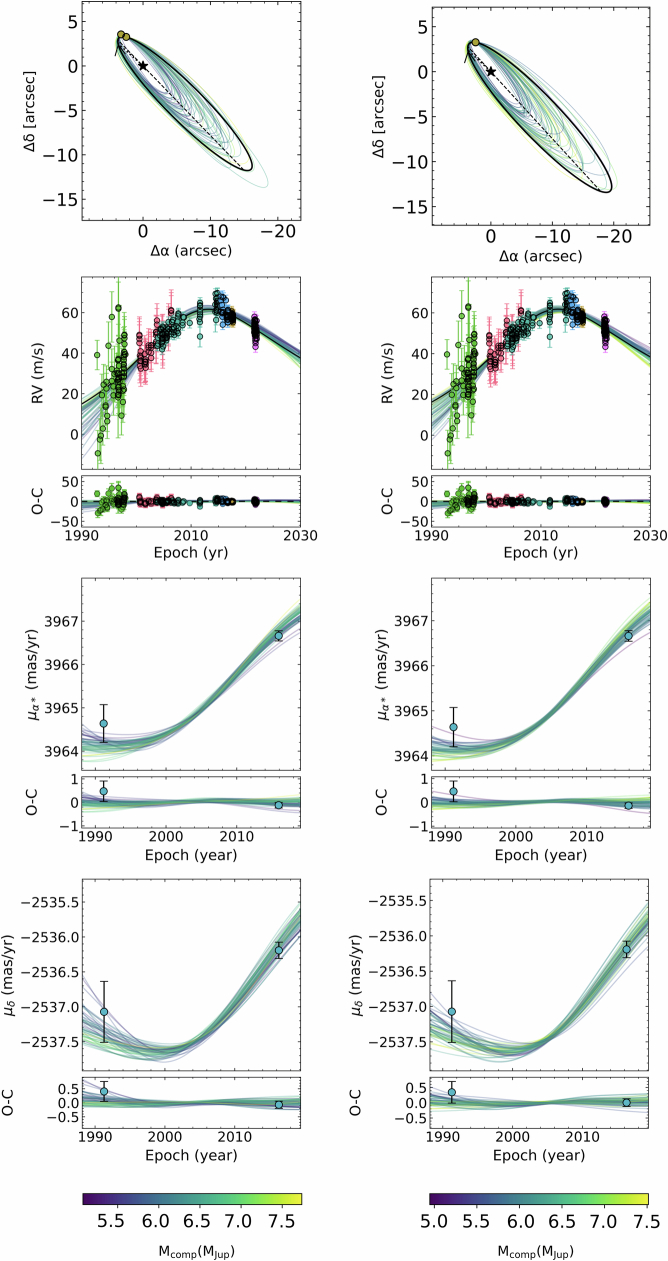
Orbit fits including RV, Hipparcos-Gaia astrometry, and both imaging points (left) or the JWST imaging only (right), assuming Eps Ind Ab is the only massive planet in the system. Rows represent (1) the on-sky orbit, (2) the RV of the target (3) the proper motion in right ascension of the host star and (4) the proper motion in declination of the host star. Orbits are very similar in both cases, and explain all in-hand data. RVs are from LC (green), VLC (pink), HARPS03 (cyan), UVES (yellow), HARPS15 (blue) and HARPS20 (pink).

We attempted to fit the orbit of the imaged planet by assuming that it is, indeed, the only planet in the system and that it is responsible for all the dynamical measurements (radial-velocity data and astrometry) of the host star. We used radial-velocity data from the long camera (LC) and very long camera (VLC) of the coudé echelle spectrometer at the European Southern Observatory (ESO)^5^, from the ultraviolet and visual echelle spectrograph (UVES)^2^ and from the high accuracy radial-velocity planet searcher (HARPS), using the data reduction presented in ref. ^54^. Eps Ind A has been targeted intensively with HARPS, and we binned any data points collected within a single night with HARPS. We treated the HARPS data as three separate instruments, to account for any possible baseline shifts during an instrument upgrade in 2015 and a telescope shutdown in 2020. This gave us 493 data points, from six independent instruments, covering from 3 November 1992 to 29 December 2021. We fitted orbits using orvara^55^, considering the 493 radial-velocity points, the two direct imaging epochs and the Hipparcos–Gaia astrometry of the host star, and using a host star mass prior of 0.76 ± 0.04 *M*_⊙_. This process converged to an orbit with mass ${6.31}_{-0.56}^{+0.60}$ *M*_J_, semimajor axis ${28.4}_{-7.2}^{+10}\,{\rm{au}}$, eccentricity ${0.40}_{-0.18}^{+0.15}$ and inclination 103.7 ± 2.3° (Extended Data Fig. 6). Without the NEAR astrometry, we found an orbit with mass ${6.17}_{-0.57}^{+0.65}$ *M*_J_, semimajor axis ${31}_{-10}^{+13}\,{\rm{au}}$, eccentricity ${0.47}_{-0.23}^{+0.15}$ and inclination 104.0 ± 2.4°. These orbits are highly consistent with each other, further validating that the NEAR epoch is a positive redetection of the companion. These orbits are also consistent with all in-hand dynamical data. Curiously, several previous works had derived properties of the claimed planet Eps Ind Ab and found consistent results^2,8,9^, but these results are inconsistent with the planet observed in this work. This may be due to overfitting of the in-hand data, as fitting accurate orbits with insufficient orbital phase coverage is notoriously hard^56^, or it may hint at another component in the system that biased the previous one-planet fits. The consistency between the fits with and without the NEAR astronomy points towards a one-planet system. We leave a detailed exploration of the discrepancy between the imaged planet and the previous radial-velocity solutions to a future work, but note that the dynamical mass derived in this work is valid only if Eps Ind Ab is the only massive planet present.

### Stellar photometry

**Extended Data Fig. 7:**
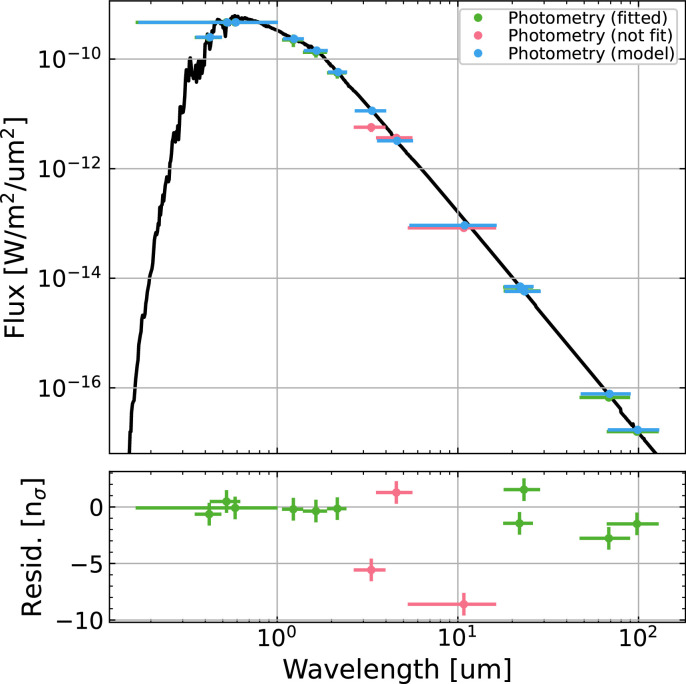
Best-fit spectrum for Eps Ind A. The spectrum is shown in black and integrated to R~100. Measured photometry is shown in green if included in the fit, and pink otherwise, while the model photometry is indicated in blue. The WISE W1, W2, and W3 channels are saturated, and in particular the W1 and W3 magnitudes^60^ are several $\sigma$ fainter than the best-fit model.

We used BT-NextGen models^57,58^ to predict the photometry of the host star Eps Ind A in the JWST/MIRI and VLT/NaCo filters. We fitted spectra to the in-hand photometry from TYCHO, Gaia, 2MASS, WISE, Spitzer/MIPS and the photoconductor array camera and spectrometer (PACS) onboard Herschel^10,47,50,59,60^. We allowed the effective temperature, log(*g*), metallicity and radius to vary and held the stellar distance fixed at 3.639 pc. We sampled the posterior with a Monte Carlo Markov chain (using emcee; ref. ^61^) to derive the best-fitting spectrum and uncertainty. The best-fitting values are effective temperature *T*_eff_ = 4,760 ± 15 K, log(*g*) = ${5.25}_{-0.34}^{+0.18}$, [Fe/H] = 0.22 ± 0.12 and *R* = 0.679 ± 0.004 *R*_⊙_, although we caution that these values are driven by photometry only, and spectroscopic fits of the stellar parameters should be used over these values when interpreting the metallicity and log(*g*) of the host star. We then derived model photometry and uncertainties by selecting samples from the Markov chain Monte Carlo (MCMC) posterior and integrating over the filter profiles. The best-fitting spectrum as well as the measured (green and pink) and model (blue) photometry of Eps Ind A is shown in Extended Data Fig. 7. We used this model to derive all companion photometry and flux limits in this work, including reconverting archival contrast curves to flux upper limits with this model.

The WISE observations of Eps Ind A appear to be unreliable. Eps Ind A is significantly saturated in the WISE W1, W2 and W3 observations (the WISE source catalogue^60^ lists saturation fractions of w1sat = 0.253, w2sat = 0.239 and w3sat = 0.144). For this reason, we did not include the W1, W2 and W3 magnitudes directly in our fitting process. Our derived stellar magnitudes show some discrepancy with those used to derive previous L′ limits^17^, which used the WISE W1 magnitude of 2.9 as a proxy for the L′ magnitude of Eps Ind A. Our stellar fit indicates a somewhat brighter magnitude for the star of 2.119 ± 0.008 and 2.115 ± 0.009 for the W1 and L′ filters respectively. The only published L-band magnitude for Eps Ind is *L* = 2.12 (ref. ^62^), consistent with our derived W1 and L′ magnitudes.

## Online content

Any methods, additional references, Nature Portfolio reporting summaries, source data, extended data, supplementary information, acknowledgements, peer review information; details of author contributions and competing interests; and statements of data and code availability are available at 10.1038/s41586-024-07837-8.

## Figures and Tables

**Extended Data Table 1:**
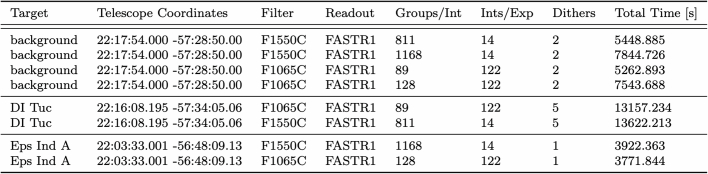
JWST/MIRI observations for Eps Ind A

All observations were carried out sequentially on 3 July 2023, and have one exposure per dither position.

**Extended Data Table 2:**
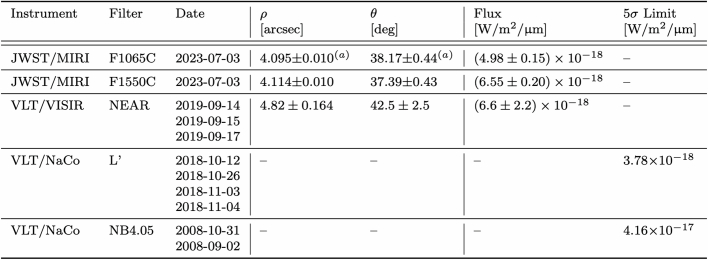
Astrometry, photometry and upper limits for Eps Ind Ab

JWST and VLT/VISIR constraints are from this work; VLT/NaCo constraints are from ref. ^17^ (L', their “special LOCI” reduction with our stellar calibration from Section 2.5) and ref. ^18^ (NB4.05, we quote a median sensitivity of the observations between 3'' and 8''). Values for multi-epoch visits (VLT/VISIR, VLT/NaCo) correspond to a combined analysis of all visits.

* a:* Note that for F1065C the star is not well centred behind the coronagraph, and the astrometry should be considered unreliable.

## Data Availability

All observational data in this work are available through public data archives. In particular, the JWST data were collected through the General Observer programme 2243 (PI E.C.M.) and are available through the Mikulski Archive for Space Telescopes (https://mast.stsci.edu). Spitzer data were collected through programmes 90 (PI M. Werner) and 41 (PI G. Rieke) and are available through the Spitzer Heritage Archive (https://irsa.ipac.caltech.edu/applications/Spitzer/SHA/). VLT data from VISIR/NEAR and SPHERE were collected through programmes 60.A-9107/103.201H (PI M. Meyer) and 110.25BR (PI E.C.M.), respectively, and are available through the ESO science archive (http://archive.eso.org/cms.html). Radial-velocity data and atmospheric models were taken from previously published literature, as indicated in the text.

## Code availability

Data reduction was carried out using the publicly available spaceKLIP package (https://github.com/kammerje/spaceKLIP), which also uses functions from the jwst pipeline (https://jwst-pipeline.readthedocs.io), the pyKLIP Python package (https://pyklip.readthedocs.io) and the webbpsf_ext package (https://webbpsf.readthedocs.io; https://github.com/JarronL/webbpsf_ext). Orbit fitting was carried out using the publicly available orvara package (https://github.com/t-brandt/orvara). We used various functions from astropy, pandas, matplotlib and seaborn in the analysis and to create figures.
